# Genetic tools discriminate strains of *Leishmania infantum* isolated from  humans and dogs in Sicily, Italy

**DOI:** 10.1371/journal.pntd.0008465

**Published:** 2020-07-24

**Authors:** Germano Castelli, Federica Bruno, Valentina Caputo, Santi Fiorella, Ignazio Sammarco, Tiziana Lupo, Antonella Migliazzo, Fabrizio Vitale, Stefano Reale

**Affiliations:** 1 National Reference Center for Leishmaniasis (C.Re.Na.L.), Istituto Zooprofilattico Sperimentale della Sicilia, Palermo, Italy; 2 Section of Dermatology, Department of Health Promotion, Maternal-Infant, Internal Medicine and Specialization of Excellence “G. D’Alessandro” (PROMISE) University of Palermo, Palermo, Italy.; 3 Tecnologie Diagnostiche Innovative, Istituto Zooprofilattico Sperimentale della Sicilia, Palermo, Italy; University of Iowa, UNITED STATES

## Abstract

**Background:**

Leishmaniasis is one of the most important vector-borne diseases and it represents a serious world health problem affecting millions of people. High levels of *Leishmania* infections, affecting both humans and animals, are recognized among Italian regions. Among these, Sicily has one of the highest prevalence of *Leishmania* infection.

**Methodology/Principal Findings:**

Seventy-eight *Leishmania* strains isolated from human and animal samples across Sicily, were analyzed for the polymorphic k26-gene and genotypes were assigned according to the size of the PCR products. A multilocus microsatellite typing (MLMT) approach based on the analysis of 11 independent loci was used to investigate populations structure and genetic diversity of the isolated strains. Six *L*. *infantum* reference strains were included in the analysis for comparison. Bayesian clustering analysis of microsatellite data showed that all the isolated strains clustered in two genetically distinct populations, corresponding to human and canine isolates respectively. A further subdivision was observed between the two main groups, giving a good correlation between human strains and their geographic origin, conversely canine population showed a great genetic variability diffused in the territory.

**Conclusions/Significance:**

Among the 78 *Leishmania* isolates, K26 analysis detected 71 samples (91%) as MON-1 zymodeme, confirming it as the predominant strain in Mediterranean area and 7 human samples (9%) as non-MON-1. MLMT gives important insights into the epidemiology of leishmaniases and allows characterization of different strains to a higher resolution than possible with zymodeme typing. Two main populations presented a strong correlation respect to the different hosts, exhibiting a co-circulation of two distinct populations of *L*. *infantum*. The population found in infected humans exhibited a correlation with geographic origin. These clusters could represent a geographically restricted population of strains with the same or related genotypes. This study can contribute to an understanding of *Leishmania* epidemiology, including the spread of reservoirs and sand fly vectors in the different foci of infection, characterizing parasites within the different hosts.

## Author summary

High levels of *Leishmania spp*. infections affecting both humans and animals are recognized among Italian regions; in particular, Sicily is an endemic area for *Leishmania infantum*. In this study 78 Sicilian *L*. *infantum* strains isolated from humans and dogs were assessed to investigate their biodiversity by genetic tools. Results were compared with 6 *L*. *infantum* reference strains included in the analysis. The evaluation of K26 genetic markers identified 91% of samples as belonging to the MON-1 zymodeme, confirming it as the predominant strain in the Mediterranean area and 9% of the samples–all isolated from humans–as non-MON-1. Multilocus microsatellite typing has proven to be a powerful tool to discriminate strains showing all the isolated strains clustered in two genetically distinct populations, corresponding to human and canine isolates, respectively. A further subdivision was observed between the two main groups, giving in the human population a correlation between microsatellite profile and geographical origin. Our results demonstrate that genetic tools are able to discriminate *Leishmania* strains and to give useful insights into the epidemiology of leishmaniasis, raising questions on the role of dogs as main reservoirs for human leishmaniasis in the Sicily region.

## Introduction

*Leishmania* is a genus of flagellate protozoa causing a broad spectrum of diseases, ranging from self-limiting localized cutaneous lesions to visceral manifestations that can arise in a fatal evolution. The disease affects people, domestic and wild animals in temperate, subtropical and tropical regions [1]. In Mediterranean areas, *L*. *infantum* is responsible for Visceral Leishmaniasis (VL) and human Cutaneous Leishmaniasis (CL) [2]. The natural cycle involves phlebotominae sandfly vectors that transmit the parasite to the vertebrate host. Epidemiology of the disease is influenced by the geographical distribution of the vectors and their host specificity. CL and VL are transmitted through a zoonotic mechanism, which involves dogs as the main *reservoir* of the infection [3]. However, many infected animals do not develop a clinical form [1], in fact a wide spectrum of lesions and clinical signs are considered characteristic for the disease (lymphadenomegaly, skin and ocular lesions, epistaxis, weight loss and signs of renal failure), known as Canine Leishmaniasis (CanL). In endemic countries the number of asymptomatic *Leishmania* infections, in immunocompetent subjects, is 5–10 times greater than the number of clinically apparent disease cases [4].

Sicily is a highly endemic region for leishmaniasis [5] due to climate conditions that facilitate vectors, the spread of parasites and life-cycle [6] represent a growing public health concern. The main form of the disease is the localized CL in the immunocompetent host. The typical clinical feature is an erythematous papule that enlarges over a few weeks to form an indolent ulcerated nodule with a hardened and raised outer border and sharply incised central crater covered by an adherent crust. The lesion usually remains localized in the inoculation site and tends to heal spontaneously within 3–6 months, leaving a hollow scar with hypo- or hyperpigmentation. Multiple lesions can occur as the result of several bites by infected sandfly. Some patients may show clinical variants of CL: eczematoid, erysipeloid, verrucous, chancriform, zosteriform, keloideal, psoriasiform. A rare localized cutaneous chronic form is lupoid leishmaniasis that, occurring over the face, may resemble discoid lupus erythematosus or lupus vulgaris. The heterogeneity of the disease manifestations observed in patients is mainly related to the species of infecting parasite and to the host’s immune response. Lesions are painless unless an additional infection develops: in this case, a regional lymphadenopathy can develop [7]. The ability of *L*. *infantum* to cause CL is also well established. MON-1 and MON-24 zymodeme strain is occasionally responsible for CL in spite of other zymodemes that mainly cause it (MON-29, MON-33, MON-78, MON-11 and MON-111) [8]; *L*. *infantum* is not a uniform species [9] even if more than 80% of the strains of *L*. *infantum* isolated in the Mediterranean area belong to zymodeme MON-1 [10,11]. The zymodeme MON-24 is the most widely distributed *L*. *infantum* dermotropic zymodeme in the Mediterranean area and it’s also one of the most common non-MON-1 zymodemes. Multilocus enzyme electrophoresis (MLEE) is the reference technique for the classification of *Leishmania as* ‘zymodemes’ with many laboratories around the world that identified different reference strains as zymodemes (with the best-known system of 'MON' types established by the National Reference Center for Leishmaniasis in Montpellier) [12]. The method is based on differences between strains in the electophoretic mobility of a set of enzymes in a potato starch gel. However, MLEE is a laborious and time-consuming method and the discrimination level is not as high as a genetic approach. In addition, the results are not homogeneous and not comparable between various laboratories [13]. Therefore, during the past years, different molecular approaches were developed to characterize isolates and to identify intra-specie polymorphism. In particular, the combined molecular tools included polymerase chain reaction-restriction fragment length polymorphism (PCR-RFLP), high-resolution melt (HRM)-based assay [14], sequencing of protein coding (i.e., gp63, hsp70, cpb) or noncoding regions (i.e., internal transcribed spacer-1, repetitive DNA sequences) [15]. Currently, microsatellites-based identification seems to be the best way to identify the circulating strains, although the introduction of the whole genome sequencing represents a revolutionary step and will lead to massive improvements.

Multilocus microsatellite typing (MLMT) represents a powerful tool useful for molecular epidemiological research, for population studies and for forensic applications. The short microsatellite sequences are highly polymorphic, codominant and dispersed throughout the genome of eukaryotic organisms. Normally, their mutation rate is 5- to 6-fold higher than that of the bulk of the DNA. The aim of this study was to investigate the genetic structure of Sicilian *L*. *infantum* populations, affecting humans and dogs, through a microsatellite based multiplex PCR assay.

## Methods

### Samples

In this study 78 *Leishmania* strains isolated from human and dogs samples between 2012 to 2014 were analyzed, comparing them with 6 reference samples of *L*. *infantum* preserved at the cryobank of National Reference Laboratory for Leishmaniasis (C.Re.Na.L., Palermo, Sicily): a MON-201 sample from dog specimen, a MON-1 (MHOM/TN/80/IPT1) sample from Tunisia, two human MON-1 strains from Sicily, one human MON-29 (MHOM/ES/81/LEM307) from Spain and one human MON-24 reference strain from Agrigento (Sicily) (Table 1).

**Table 1:** Reference strains and characteristics of *Leishmania*-positive samples included in the study.

**Reference Strains**								
**ID**	**Zymodeme**		**Host**		**Geographic origin**			
**1**	MON-1		Human			Tunisia		
**2**	MON-24		Human			Agrigento (Sicily)		
**3**	MON-29		Human			Spain		
**4**	MON-201		Dog			Messina (Sicily)		
**5**	MON-1		Human			Palermo (Sicily)		
**6**	MON-1		Human			Agrigento (Sicily)		
**Humans**			**Dogs**					
**ID**	**City/Province**	**VL/CL** **(Lesion localization)**	**ID**	**City/Province**	**ID**	**City/Province**	**Leishmaniasis**	
**7**	Licata/ Agrigento	VL (Visceral)	**35**	Palermo	**63**	Catania	
**8**	Ciminna/ Palermo	CL (Right forearm)	**36**	Palermo	**64**	Catania	
**9**	Agrigento	CL (Left forearm)	**37**	Palermo	**65**	Catania	
**10**	Caccamo/ Palermo	CL (Left forehead)	**38**	Palermo	**66**	Catania	
**11**	Caccamo/ Palermo	CL(Left forehead)	**39**	Palermo	**67**	Catania	
**12**	Sciacca/ Agrigento	CL (Right arm)	**40**	Palermo	**68**	Catania	
**13**	Racalmuto/ Agrigento	CL (Right cheek)	**41**	Palermo	**69**	Catania	
**14**	Racalmuto/ Agrigento	CL (Left zygomatic region)	**42**	Palermo	**70**	Catania	
**15**	Canicattì/ Agrigento	CL (Left arm)	**43**	Palermo	**71**	Catania	
**16**	Baucina/ Palermo	CL (Right hand)	**44**	Palermo	**72**	Catania	
**17**	Chiusa Sclafani/ Palermo	CL (Left leg	**45**	Palermo	**73**	Catania	
**18**	Agrigento/ Agrigento	CL (Left submandibular region)	**46**	Palermo	**74**	Trapani	
**19**	Sciacca/ Agrigento	CL (Left arm)	**47**	Palermo	**75**	Agrigento	
**20**	Caltabellotta/ Agrigento	CL (Left arm)	**48**	Palermo	**76**	Trapani		CanL
**21**	Caltabellotta/ Agrigento	CL (Left arm)	**49**	Palermo	**77**	Trapani	
**22**	Sciacca/ Agrigento	CL (Left zygomatic region)	**50**	Palermo	**78**	Trapani	
**23**	Riesi/ Caltanissetta	CL (Right arm)	**51**	Palermo	**79**	Trapani	
**24**	Caltanissetta/	CL (Right arm)	**52**	Palermo	**80**	Trapani	
**25**	Collesano/ Palermo	CL (Right temporal region)	**53**	Palermo	**81**	Trapani	
**26**	Bompensiere/ Caltanissetta	CL (Right elbow)	**54**	Palermo	**82**	Trapani	
**27**	Palermo	CL (Right cheek)	**55**	Palermo	**83**	Modena	
**28**	Agrigento	CL (Right wrist)	**56**	Messina	**84**	Reggio Calabria	
**29**	Sciacca/ Agrigento	CL (Right leg)	**57**	Messina			
**30**	Ciminna/ Palermo	CL (Right Eyebrow)	**58**	Messina			
**31**	Roccapalumba/ Palermo	CL (Right leg)	**59**	Catania			
**32**	Licata/ Agrigento	CL (Left leg)	**60**	Catania			
**33**	Burgio/ Agrigento	CL (Right leg)	**61**	Catania			
**34**	Chiusa Sclafani/ Palermo	CL (Right zygomatic region)	**62**	Messina			

VL, Visceral leishmaniasis; CL, Cutaneous Leishmaniasis; CanL, Canine Leishmaniasis.

Twenty-eight human patients showing forms of CL were examined at the Section of Dermatology of University of Palermo; on the other hand, the patients affected by VL (immunocompromised patient HIV+) were examined at Infectious Disease Department of University of Palermo. Human lesion is usually an erythematous papule, slightly itchy, slow growing, which subsequently progresses into a nodule, then an ulcer, over a few weeks/months. Leishmaniasis ulcers are usually asymptomatic, granulomatous based, with erythematous and raised margins, covered with crusts. Painful lesions indicate over infection. Fifty samples of lymph node aspirates from canine species affected by CanL: 48 coming from different Sicilian provinces and 2 from different Italian region (Modena and Reggio Calabria) were included in this study.

### Parasitological examination

We evaluated *Leishmania* infection through May-Grunwald-Giemsa method, real time PCR [1,16] and parasite isolation. Parasite isolation was performed from several samples: skin scrapes for CL, bone marrow for VL and lymph node aspirates for CanL. *Leishmania* parasites were cultivated in RPMI-PY medium [17] supplemented with 10% heat-inactivated fetal bovine serum (FBS) at 25°C for up to 30 days.

### DNA extraction and sequencing

The cultivated strains were harvested, washed twice with NaCl 0.3%, and centrifuged. The total DNA was extracted from the promastigotes as follows: the pellet was lysed by heating at 96°C for 20 minutes with 400 μl of a mixture containing 1% Tween 20 (Sigma, St. Louis, MO, USA), 1% Nonidet P-40 (Sigma) and 20% Chelex resin (Bio-Rad, Hercules, CA, USA) prepared in sterile distilled water. The mixture was centrifuged at 14000 g for 10 minutes at 4°C and the DNA-containing upper phase was then collected [10,16] and stored at -20°C until used. All samples in the study were sequenced for the Internal Transcribed Spacer-1 (ITS-1) region for species identification according to El Tai et al. [18]. Moreover, a repeat region of the hydrophilic acilated surface protein B (HASPB) gene, also known as k26 gene, was sequenced [19]. The estimation of the k26-PCR product size was determined by capillary electrophoresis on a TapeStation 2200 and High Sensitivity D1000 ScreenTape kit (Agilent Technologies). The genotypes were assigned according to the size of the PCR products and adjusted considering the gene size variability, due to the number of 39/42 repeated nucleotide motifs [20, 21].

### Microsatellite analysis

Microsatellites are neutral markers in non-coding regions of the *Leishmania* genome and can contributes to a better understanding of the geographical distribution, dynamics of *Leishmania* populations and epidemiology of the disease. A panel of 11 microsatellite loci [22] were simultaneously typed to investigate the genetic polymorphisms within the *L*. *infantum* strains isolated from human and animal samples across Sicily. This Microsatellite panel, tested on the 84 *L*. *infantum* strains, were combined in multiplex with different fluorophores (Dye) according to the annealing temperatures of their primers (Table 2).

**Table 2:** Microsatellite loci with corresponding primers sequences [22] and annealing temperatures.

Locus	Chromosome	Dye	Forward primer (5’-3’)	Reverse primer (5’-3’)	TA (°C)
Li41-56	36	FAM	TTGCTTCATGATAACAACTTGG	CCTGTTGGTGTGAGTTCGTG	50
Li46-67	31	HEX	TCTTCTTTCGTTAGCTGAGTGC	CTGTATCACCCATGAGGGGC	
Li71-7	ND	FAM	GCTGCAGCAGATGAGAAGG	GTGAGAAGGCAGGGATTCAA	
Li71-33	31	HEX	CTCCTTTCACACCGCCTCT	GAGAGAAGACGAGCCGAAGT	
Li22-35	ND	FAM	CTTGATGTTCGGGTTAGCAAGT	ATGCACACCAAAAATCATGTG	52
Li23-41	25	HEX	GATCGGAGGTGACAGCGT	CCTTTAACTGCCAGTGCG	
Li21-34	1	NED	GAGAAAGCAAGACACGAGATGA	GAGGCGTTTTCCTTCTGGTAG	
Li45-24	16	FAM	GCGCCTACAGGCATAAAGGA	CTGGCGCATCAACGGTGT	
Lm2TG	1	NED	AAAAAGCGAGGAATGAAAGAA	TCCCTCCCCTCTACAACCTT	
Lm4TA	1	HEX	TTTGCCACACACATACACTTAG	GTAGACGACATCGCGAGCAC	
Li715/2	35	NED	GCACGGTCGGCATTTGTA	GATAAACGAGATGGCCGC	56
TubCA	34	FAM	GGCGTGGTTGCTAAACTGAT	GCCTGCGCACACAGAGAC	

ND, not determined; TA, annealing temperature.

The multiplex PCR assay was carried in triplicate for each sample in a volume of 20 μl containing 1X Type-it amplification mix reaction buffer (Qiagen), 0.5 μM of each marked primer and 20 ng of DNA template. After an initial denaturation step of 4 minutes at 94°C, the samples were processed through 30 cycles consisting of 50 s at 94°C, 40 s at the annealing temperature (TA) indicated in Table 2, and 1 minute at 72°C, followed by a terminal elongation step of 4 minutes at 72°C [22]. The primers sequence were published by Ochsenreither et al.[22]. Housekeeping tubulin gene TubCA was added to assess the efficiency of the microsatellite assay. The amplicons were diluted ten times in water and 2 μl of each were added to 11 μl of the injection mix containing formamide and ROX 500 (Thermo) as internal size standard, according to the microsatellite protocol for AB3130 sequencing analyser (Thermo). Amplicons were then separated based on their molecular size and the raw data were analyzed by GeneMapper Software v.3.7 using a specific bin sets manager to size all the allele within the frames including each locus.

### Genetic diversity, phylogenetic and clustering analyses

To estimate genetic diversity and resolution power of the selected microsatellite panel, GenAlEx 6.5 was adopted [23]. The following population genetics parameters were evaluated: number of alleles per locus (Na), number of effective alleles (Ne), allele size range and allele frequencies, observed (Ho) and expected (He) heterozygosity [24, 25], and fixation index (Fst) [26]. The Bayesian assignment approach implemented in Structure v. 2.3.3 [27], and based on pattern of allele frequencies from MLMT profiles, was used to estimate the number of distinct genetic clusters within samples. Number of clusters (K) varying from 1 to 10 was employed using admixture model and correlated allele frequencies, for 500,000 generations, with a burnin of 50,000 and 10 iterations. Best K value was identified by Structure Harvester ver. 0.6.94 [28]. Data were sorted by CLUMPP [29], and shown as bar-plot diagram by Distruct [30]. The Evanno method [31] was employed to detect how many clusters of individuals are expected by the allelic distribution in the sample, and how individuals are distributed among those clusters. We evaluated the length of burnin, as well as the stabilization of the parameters of the analysis using the plot function present in Structure software Phylogenetic analysis was performed based on MLMT profiles by means of Microsatellite Analyzer (MSA 4.1) that was employed to generate a pairwise distance matrix between each of the samples using Nei's chord distance algorithm [24]. This matrix was then used to plot a phylogenetic tree by TreeDyn 198.3 (http://www.treedyn.org/).

### Ethical statement

Human Samples were collected for diagnostic purposes, were stored at the cryobank of C.Re.Na.L. was carried out at the Dermatology Unit, Department of Health Promotion, Maternal-Infant, Internal Medicine and Specialization of Excellence “G. D’Alessandro” (PROMISE) University of Palermo (Palermo, Italy), while MLMT evaluation was performed at the Istituto Zooprofilattico Sperimentale della Sicilia—C.Re.Na.L. (Palermo, Italy). Samples were coded and anonymized. All adult subjects provided written informed consent, and a parent or guardian of any child participant provided informed consent on their behalf. All animal samples were obtained for diagnostic purpose with no unnecessary invasive procedures including parasitological confirmation of CanL. Oral informed consent was obtained from the owners of dogs at the time of clinical examination.

## Results

The ITS-1 sequences obtained for the 84 samples included in this study showed very high similarity (from 94.3% to 97.5% of identity) with a sequence previously identified as *L*. *infantum* (GenBank Acc Number KM677134.1). Capillary electrophoresis detected different PCR products of different sizes for the polymorphic K26 gene. In detail, all canine K26 genes showed a fragment size of 626 bp; while human samples presented different amplicon sizes: 626 bp (21 samples), 680 bp (1 sample), 385 bp (6 samples) (Table 3).

**Table 3:** Amplicon sizes of K26 gene by PCR assay of *Leishmania infantum* strains studied.

Host	Samples number	K26 size	Samples IDs
Dogs	50	626 bp (MON-1)	35–84
Humans	28	626 bp (MON-1)	7, 8, 10, 11, 12. 13, 14, 15, 18, 19, 20, 21, 22, 23, 25, 27, 28, 29, 31, 33, 34
		680 bp (non-MON-1)	16
		385 bp (non-MON-1)	9, 17, 24, 26, 30, 32

Reference strains showed K26 sizes corresponding to their MLEE profiles, in particular MON-1 strains (1, 5 and 6) showed a 626 bp size, whereas the non-MON-1 strains (2, 3 and 4) showed different amplicon sizes. MLMT analysis revealed that all evaluated microsatellite loci of *L*. *infantum* strains were polymorphic. The number of effective alleles (Ne) value differed from the Na value, ranging from 1.738 (Li2134) to 7.254 (Li2235) alleles per locus with an average of 3.347. Li4156, Li2235 and Li4524 showed the highest level of heterozygosity (0.778), whereas the Li717 marker showed the lowest (0.148) (Table 4).

**Table 4:** Population genetics data based on chromosomal microsatellites tested in *Leishmania infantum* samples from Sicily.

Locus	Na	Ne	Ho	He	Fst
***Li4156***	6	5.063	0.778	0.802	0.330
***Li4667***	5	3.664	0,423	0.727	0.315
***Li717***	4	2.150	0.148	0.535	0.427
***Li7133***	5	3.431	0.333	0.709	0.477
***Li2235***	10	7.254	0.778	0.862	0.382
***Li2341***	5	2.522	0.074	0.604	0.276
***Li2134***	5	1.738	0.370	0.425	0.355
***Li4524***	4	2.518	0.778	0.603	0.509
***Lm2TG***	3	2.219	0.667	0.549	0.569
***Lm4TA***	8	3.109	0.667	0.678	0.464
***Li7152***	7	3.149	0.519	0.682	0.358
**Mean**	**5.63**	**3.34**	**0.503**	**0.652**	**0.406**

Na, Number of alleles; Ne, Number of effective alleles; Ho, Observed heterozygosity; He, Expected heterozygosity; Fst, Fixation index.

**Fig 1:**
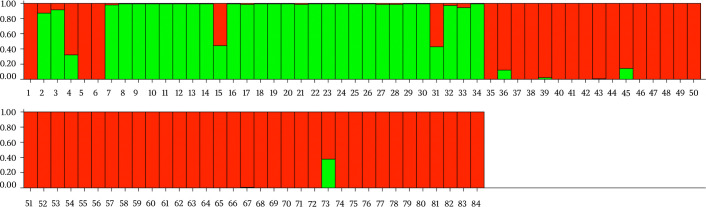
Estimated population structure for the 84 *Leishmania infantum* strains isolated from Sicily (south Italy), as inferred by STRUCTURE on the basis of microsatellite profiles. Each individual is represented by a single vertical line. Each color represents one population: green for POP-A and red for POP-B. The length of the colored segment shows the strain’s estimated proportion of membership (Q) in that population. Sample numbers are according to Table 1.

The mean Ho and He values were 0.503 and 0.652 respectively. Li4156, Li2235 and Li4524 markers showed the highest level of Ho (0.778), whereas the Li717 marker showed the lowest (0.148). The population showed a significant deficiency in the Ho value compared to the He according to the Hardy Weinberg Equilibrium (HWE) (Table 4). Clustering analysis data seems to confirm previous data of Rugna et al. [32], who had observed a host-related structure within populations of *L*. *infantum* strains from northeastern Italy differentiating two main populations, one mainly detected in dogs and the other detected in humans. In our samples we identified two distinct populations, human (POP-A) and dog (POP-B) that grouped species-specific strains. Population POP-A consisted of 24 samples from human patients affected by CL, one sample affected by VL (ID: 7) and 2 reference strains (ID: 2 and 3) typed respectively as MON-24 and MON-29. Population POP-B comprised all of the *L*. *infantum* strains isolated from canine species and three reference strains (ID: 1, 5 and 6) isolated in humans and previously identified as MON-1 zymodeme. Samples 15 and 31 from human and 73 from dogs showed admixed microsatellite alleles profiles between POP-A and POP-B (Fig 1).

**Fig 2:**
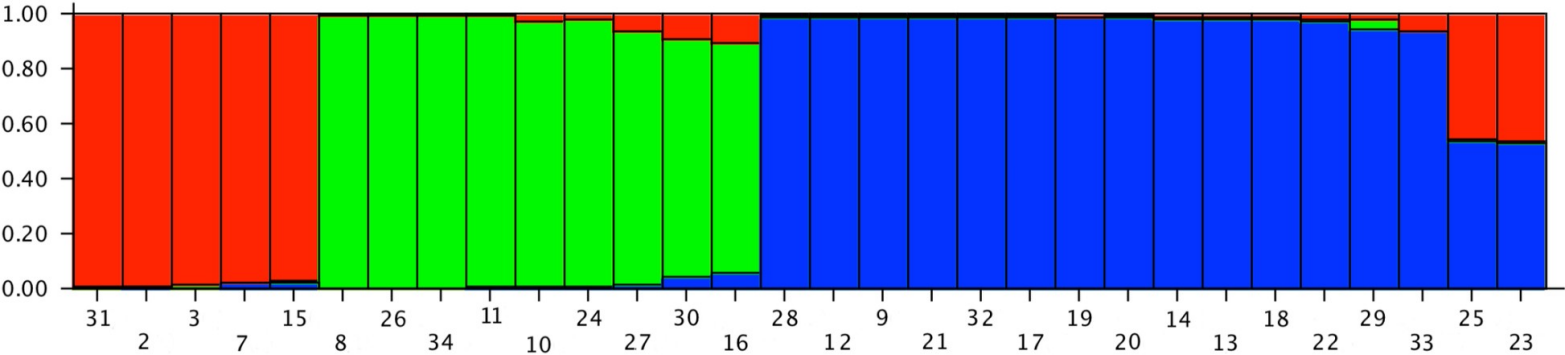
Estimated population structure for human isolates of *Leishmania infantum* (POP-A). Three main subpopulations are present. Red subpopulation (POP-A1) grouped reference strains 2 and 3, together with ID samples 15, 31 and 7; green subpopulation (POP-A2) grouped samples mainly from the north of Sicily while blue cluster (POP-A3) grouped samples mainly from southern area of the island.

**Fig 3:**
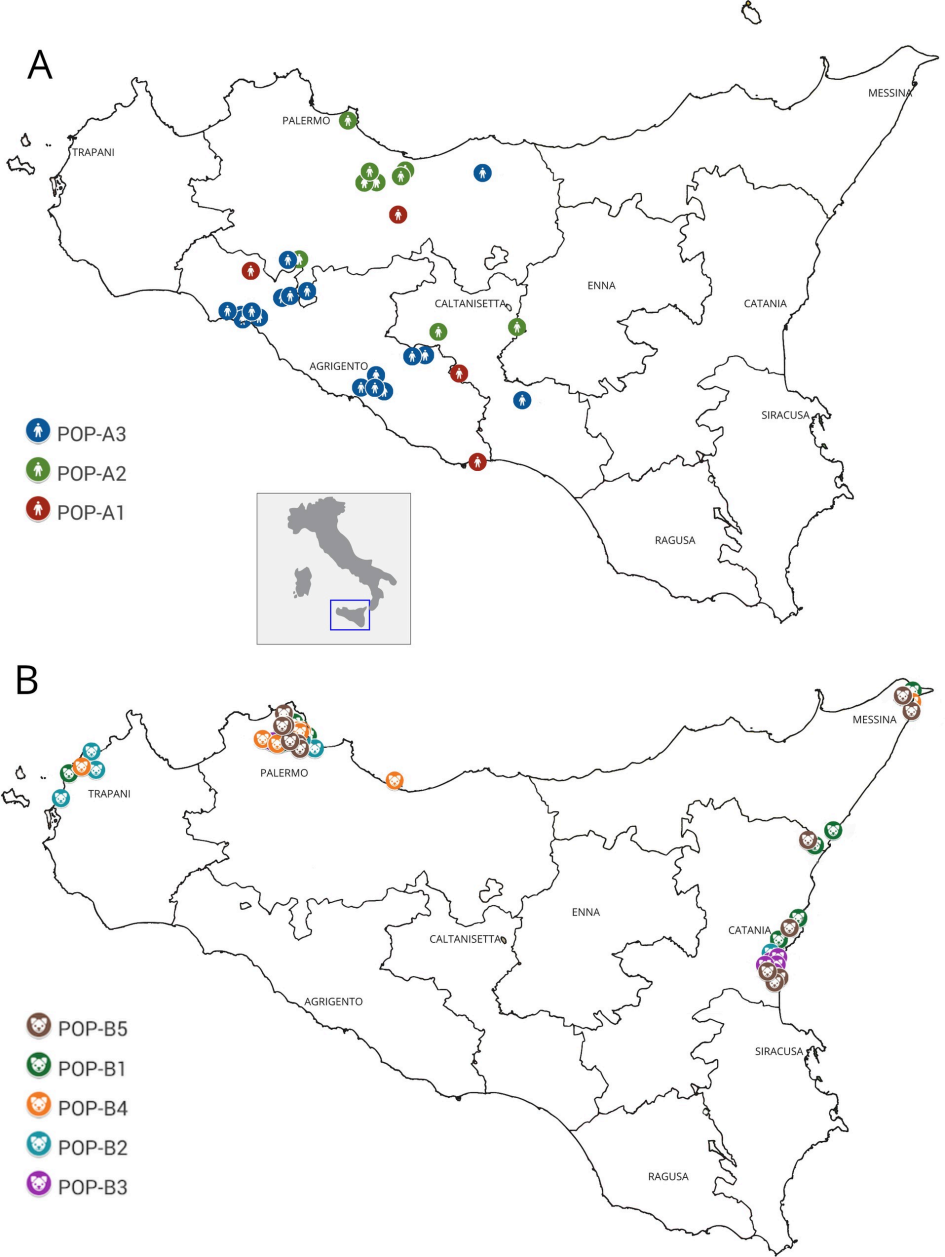
Geographical distribution of 78 *Leishmania* strains isolated from Sicily region (south Italy). **(A)** Distribution of human strains belonging to POP-A. Colors depict the subpopulations inferred by STRUCTURE analysis: red for POP-A1, green for POP-A2 and blue for POP-A3. **(B)** Distribution of canine strains belonging to POP-B. Colors depict the subpopulations inferred by STRUCTURE analysis: green for POP-B1, light blue for POP-B2, purple for POP-B3, orange for POP-B4 and brown for POP-B5.

The populations POP-A and POP-B (humans and dogs) were then, separately re-analyzed by Structure. This second clustering analysis revealed the existence of three sub clusters in humans to POP-A1, POP-A2 and POP-A3) and five subclusters in dogs (POP-B1, POP-B2, POP-B3, POP-B4 and POP-B5). The POP-A1 grouped reference strains 2 and 3, together with sample IDs: 15, 31 and 7 (the unique VL sample). The POP-A2 grouped all samples coming from the north of Sicily (Palermo area), while POP-A3 grouped *Leishmania infantum* isolated from southern part of the island (Agrigento) (Figs 2 and 3A); moreover, within this cluster two human strains showed an admixture profile with POP-A2.

**Fig 4:**
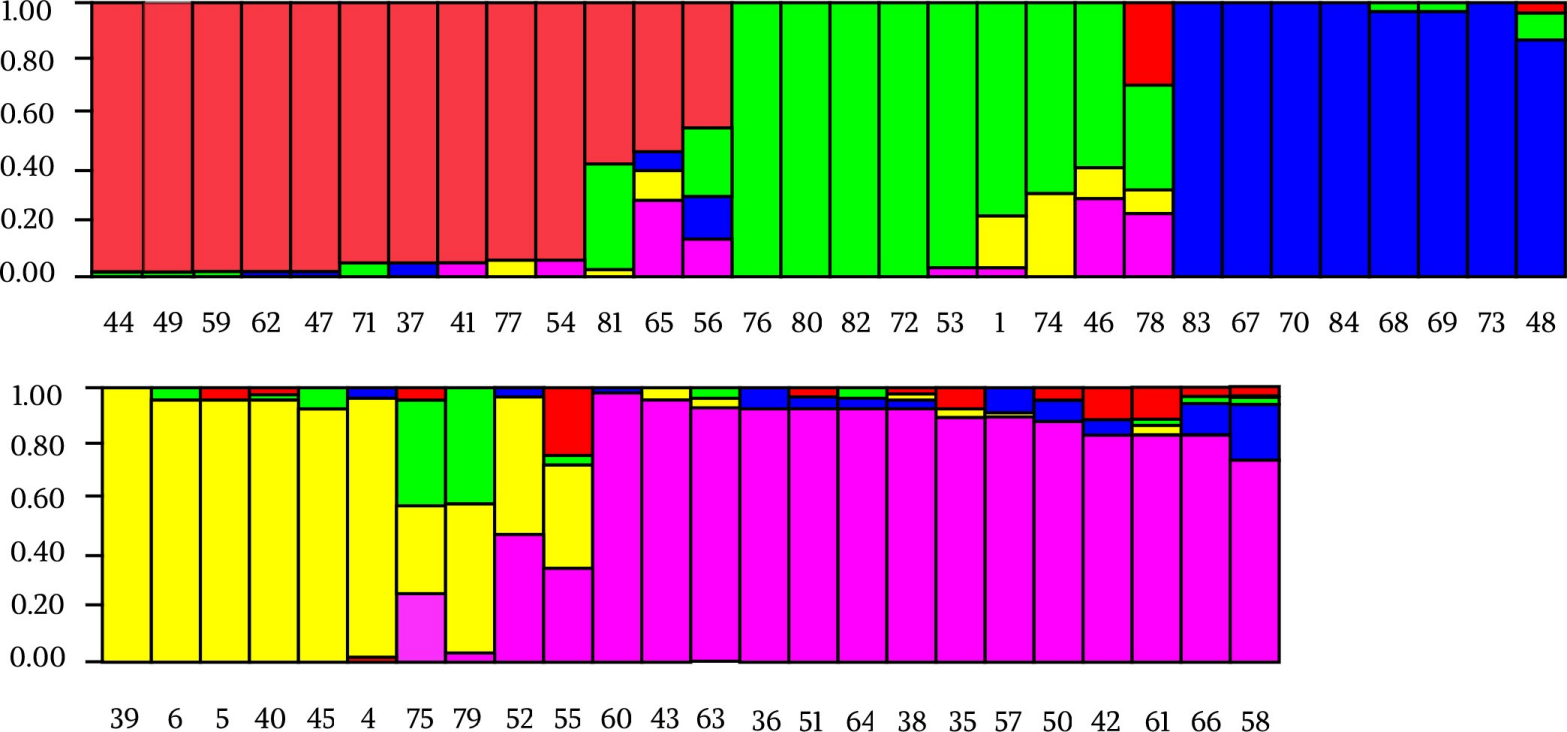
Estimated population structure for canine isolates of *Leishmania infantum* (POP-B). Five main subpopulations are present. Each color represents one population and the length of the colored segment shows the strain’s estimated proportion of membership (Q) in that population. Colors represent the subpopulations inferred by STRUCTURE analysis: red for POP-B1, green for POP-B2 included 5 of the 8 strains in the province of Trapani, blue cluster for POP-B3 grouped canine samples mainly from Catania province, together with extra regional IDs 83 and 84, yellow for POP-B4 grouped Palermo province samples population together with three reference strains (ID: 6, 5 and 4) and purple for POP-B5.

The canine cluster (POP-B) showed a higher genetic differentiation than its human counterpart differentiating into 5 sub-populations. In particular, POP-B2 included 5 of the 8 strains from the province of Trapani. The majority of samples of POP-B3 came from Catania province (5/6), together with the extra regional strains (ID: 83 and 84). More samples of Palermo province (5/9) clustered in POP-B4 together with three reference strains (ID: 6, 5 and 4). POP-B1 and POP-B5 populations showed a geographical distribution widespread across three provinces (Palermo, Messina and Catania) (Figs 3B and 4).

**Fig 5:**
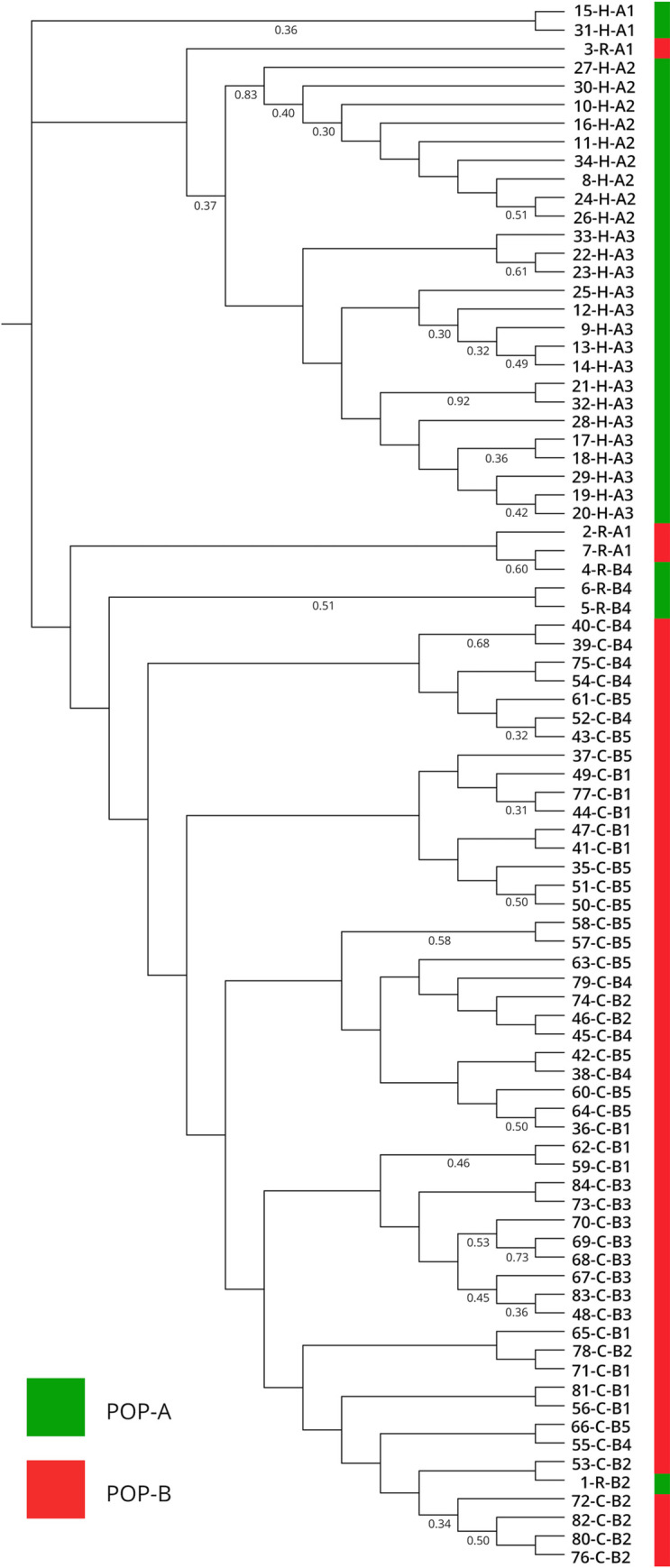
Phylogenetic tree of 84 *Leishmania* strains obtained by Nei's chord distance algorithm [24]. Samples are differentiated by the color of STRUCTURE designation (K = 2): POP-A samples in green, POP-B samples in red. Strains designations specify, respectively the ID code, the hosts (H, human; C, canine; R, reference strains) and sub-populations defined by STRUCTURE analysis.

The phylogenetic method based on a matrix of Nei’s distances was used to confirm the populations derived from Structure, including all 78 samples of *Leishmania infantum* and 6 reference strains, confirms Bayesian clustering with K = 2, showing two main monophyletic clades. The first clade included all canine samples as inferred by Structure software. The second clade grouped all human and 2 dog samples (ID: 35, 46) (Fig 5).

## Discussion

Leishmaniasis is an endemic disease widespread in many areas of the Mediterranean basin. *Leishmania infantum* is the predominant species in Sicily and the increase in this infection represents an emerging health problem for humans. An epidemiological surveillance system would be important to discriminate the different strains around the sicilian region, by identifying the infection foci and studying the geographic distribution of the parasite. The structure of the *Leishmania* population in Sicily has been characterized by using different approaches. For this purpose, strains isolated from both humans and dogs living in different Sicilian provinces were analyzed. The first step was to identify the species of the 78 *Leishmania* strains isolated from affected patients by the amplification of the ITS marker. The sequences obtained showed a strong correspondence with different strains of *L*. *infantum* from the Mediterranean basin, as reported by Kuhls et al. [33], confirming that this species is endemic in the Sicilian area [32, 5].

A short nuclear repetitive region (k26 gene) was studied to differentiate the *Leishmania* samples at strain level. According to previous studies [19,34] the 626 bp size was found only in MON-1 zymodeme, highly prevalent in Sicily [35]. So, K26-PCR assay was applied to distinguish MON-1 from non-MON-1 strains. All reference MON-1 strains showed a single amplicon size of 626 bp for k26 gene, confirming the correspondence between the MLEE analysis and the k26 typing as found by Haralambous et al [19]. Among the 78 isolates, capillary electrophoresis detected: 71 samples (91%) as MON-1 zymodemes, confirming the latter as the predominant strain in the Mediterranean area [36] and seven samples (9%) as non-MON-1. In particular, the non-MON-1 strains were isolated from humans and showed amplicon sizes of 385 and 680 bp. The product size of 680 bp was previously described in *Leishmania* strains isolated from patients residing in Emilia Romagna region (Italy) [15] and the other amplicon size (385 bp) was previously obtained in *L*. *infantum* strain from Malta and Sicily [19]. Multilocus microsatellite typing (MLMT) gives important insights into the epidemiology of leishmaniases and allows characterization of different strains to a higher resolution respect to zymodeme typing.

The two main populations detected (POP-A and POP-B) presented a strong correlation with the different hosts. In particular POP-A grouped all human derived isolates while POP-B was exclusive for canine samples, exhibiting a host related genetic structure. The clustering analysis showed some exceptions, in particular samples 15 and 31, belonging to human population (POP-A), presented an admixed microsatellite profile, with genetic characteristics of both human and canine samples. These hybrid accessions could be explained with coinfection and inter-species crosses and subsequent gene flow between canine and human MON-1 strains [32]. Strains with mixed genotypes are most likely products of genetic cross between strains of the POP-A and POP-B populations (Fig 1). The 15 and 31 strains had an unusual high number of heterozygous loci with alleles characteristic for both the populations and was assigned to intermediate positions between these two populations (S1 Table). Since multiple heterozygous loci were identified among hybrids and gene flow was detected between POP-A and POP-B populations, recombination between strains with different alleles seems to be the most plausible explanation [37]. Strains with mixed genotypes are also frequently observed [38] and the mechanisms underlying hybrid formation are, however still unknown. As *L*. *infantum* infects humans and dogs and continues its life cycle in the sandfly midgut, it is vital to investigate the compartments where genetic recombination of *Leishmania* could take place [39]. The observation of a higher number of putative recombinant genotypes in the vector could suggest that recombination occurs in the vector [33]. Akopyants et al. [40] provided evidence that the invertebrate stages of *Leishmania* are capable of having a sexual cycle consistent with a meiotic process in the sand fly vector, and hybrids were transmitted to the mammalian host by sand fly bite. Whether these strains were hybrids, aneuploids or simply mixtures of two or more different populations of parasites needs to be clarified by cloning the parasites and sequencing additional genomic targets. Furthermore, Bayesian clustering of POP-A confirmed a good correlation between human strains and their geographic origin. In fact, samples from sub-population POP-A2 were patients coming from central or northern part of Sicily (Palermo and Caltanissetta provinces), while samples from sub-population POP-A3 came from central or southern, in particular Agrigento and southern of Palermo province as shown in Fig 3. These clusters could represent a geographically restricted population of strains with the same or related genotypes.

MLMT allows characterization of strains from different geographical areas and gives important insights into the epidemiology of disease and dynamics of *Leishmania* populations [41]. As being neutral markers in non-coding regions of the genome, the clinical manifestations are not necessarily linked to MLMT profiles [42]. Conversely, canine population (POP-B) showed a great genetic variability. Five sub-populations with a widespread geographical distribution along the investigated Sicilian provinces, (Palermo, Agrigento, Caltanissetta, Messina and Catania) were detected. No correlation between identified clusters and geographical distribution was observed, including two canine samples 83 and 84 coming from other Italian regions, which exhibited a similar microsatellite profile to strains isolated from Sicilian dogs (Fig 4). However, these two dogs with their owners had traveled in Sicily previously and we cannot exclude a transmission of the infection from the Sicilian endemic region. The genetic variability in canine strains is determined by the prevalence of Leishmaniasis in sicilian territory [5]. Therefore, high genetic variability in canine strains could be caused by different *Leishmania* strains co-infections, since dogs are a necessary *reservoir* for the biological cycle of *L*. *infantum* unlike the human which is an occasional host [43].

There are few studies that have explored the genetic diversity in the canine strains in other Italian regions, Rugna et al. [32] observed that observed that the population of parasites present in dogs in Emilia Romagna showed the highest levels of genetic diversity and allelic richness, and proposed that this population might have been introduced from other Italian regions due to active reservoir migration. In Sicily, all canine isolates showed a substantial genetic diversity probably due to a more rooted endemicity of *Leishmania* over time, an increased number of stray dogs, an increase in tourist flows to the island and consequent dogs movements.

Phylogenetic analysis supported the Bayesian differentiation between the two clusters, including the POP-A and POP-B samples in two monophyletic groups (Fig 5), confirming the presence of two *Leishmania* populations among dogs and humans respectively. The identified clades on the phylogenetic tree did not correspond to the 8 total subclusters resulted from the second Bayesian analysis. This is in agreement with the observation that microsatellite data clustering methods proved to be superior to distance-based approaches for the processing of data sets with low variability [32]. Obtained data showed an association between parasite genotypes and respective hosts; in particular a good correlation between the MLMT profiles and geographical distribution of *Leishmania* strains were found in human host.

The presence of *L*. *infantum* sand fly vectors and stable parasite circulation in Sicily [5, 44] highlights the necessity to conduct a comprehensive entomological and epizootic survey for the identification of *Leishmania* vectors and the isolation of *Leishmania* parasites from sand flies. This would give the opportunity to thoroughly investigate the population structure of *L*. *infantum* and disease dynamics in Sicily.

The genetic variability detected in this study discriminated two different host-specific populations. Human derived isolates showed genetic polymorphism related to different Sicilian provinces. Canine isolates, in contrast, showed a great genetic diversity but uniformly distributed in the entire island. It could be possible that human isolates are not related to canine *reservoir* but future studies on isolates from other mammals and from the vectors as well, are necessary to address this topic. These findings shed light on the transmission dynamics of parasites and the combined molecular tools can constitute a helpful support for parasite tracking and for a better understanding of the epidemiological evolution of leishmaniasis.

## Supporting information

S1 Table Characteristics of reference strains, *Leishmania*-positive samples and MLMT profiles included in the study. VL, Visceral leishmaniasis; CL, Cutaneous Leishmaniasis; CanL, Canine Leishmaniasis; Pop, population.
(XLSX)
